# Cluster of an Unusual Amnestic Syndrome — Massachusetts, 2012–2016

**DOI:** 10.15585/mmwr.mm6603a2

**Published:** 2017-01-27

**Authors:** Jed A. Barash, Nick Somerville, Alfred DeMaria

**Affiliations:** ^1^Lahey Hospital and Medical Center, Burlington, Massachusetts and Soldiers’ Home, Chelsea, Massachusetts; ^2^Epidemic Intelligence Service, CDC; ^3^Massachusetts Department of Public Health, Jamaica Plain, Massachusetts.

In November 2015, a neurologist in the Boston, Massachusetts, area reported four cases of an uncommon amnestic syndrome involving acute and complete ischemia of both hippocampi, as identified by magnetic resonance imaging (MRI), to the Massachusetts Department of Public Health (MDPH) (*1*). A subsequent e-mail alert, generated by the Massachusetts Board of Registration in Medicine and sent to relevant medical specialists (including neurologists, neuroradiologists, and emergency physicians), resulted in the identification of 10 additional cases that had occurred during 2012–2016. All 14 patients (mean and median age = 35 years) had been evaluated at hospitals in eastern Massachusetts. Thirteen of the 14 patients underwent routine clinical toxicology screening at the time of initial evaluation; eight tested positive for opioids, two for cocaine, and two for benzodiazepines. Apart from sporadic cases (*2*–*6*), this combination of clinical and imaging findings has been reported rarely. The apparent temporospatial clustering, relatively young age at onset (19–52 years), and associated substance use among these patients should stimulate further case identification to determine whether these observations represent an emerging syndrome related to substance use or other causes (e.g., a toxic exposure).

The four patients reported in November 2015 had been evaluated at a single Boston-area medical center during the preceding 3 years (*1*). MRI of the head revealed changes consistent with acute and complete ischemia of both hippocampi (Figure) in all four patients at the time of initial evaluation. Three of the four patients tested positive for opiates on initial toxicology screening, and the fourth, who was not tested, had a reported history of heroin use. No readily apparent evidence for another established etiology of hippocampal amnesia (*7*,*8*) existed for any of the patients. Several previous isolated case reports were associated with cocaine use only (*2*–*4*), and one case of complete unilateral hippocampal infarction involving heroin inhalation was reported in France in 2013 (*9*).

**FIGURE:**
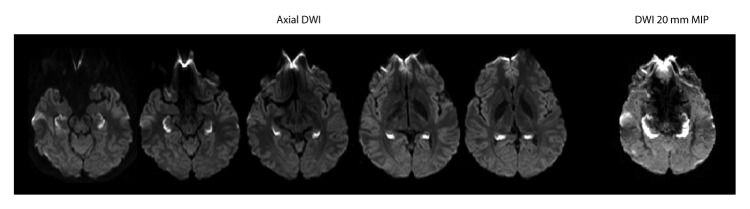
Diffusion-weighted imaging (DWI)* findings at initial brain MRI in a patient with unusual amnestic syndrome — Massachusetts, 2012 **Abbreviations:** MIP = maximum intensity projection; mm = millimeter; MRI = magnetic resonance imaging. * Axial DWI demonstrates bright signal consistent with complete bilateral hippocampal ischemia. The complete extent of hippocampal ischemia is best evident on thick 20 mm MIP images constructed from the axial DWI data.

**Source:** Adapted from: Small JE, Butler PM, Zabar Y, Barash JA. Complete, bilateral hippocampal ischemia: a case series. Neurocase 2016;22:411–5. Reprinted with permission of the publisher, Abingdon, (OX), United Kingdom: Taylor & Francis, Ltd. http://www.tandfonline.com.

In February 2016, MDPH requested that neurologists, radiologists (including neuroradiologists), and emergency department physicians report any similar cases for medical record review. For the purposes of the review, a case was defined as a patient evaluated in Massachusetts since January 1, 2012 with 1) new onset amnesia in the absence of evidence to support a readily apparent cause, and 2) changes consistent with acute and complete ischemia of both hippocampi on MRI at initial assessment. To investigate each report, the authors (including a board-certified neurologist) reviewed available clinical documentation and diagnostic testing. After preliminary case review, demographic, behavioral, and clinical data, including information related to substance use, were abstracted for analysis from records of patients who met the case definition.

Including the four initial cases, medical records of 25 patients, dating back to 2008, were reviewed after the February 2016 request by MDPH for case reporting. Medical testing was not uniform among all patients, because each patient underwent variable and extensive testing based on clinical context and the assessment of their health care provider. Fourteen (56%) patients met the case definition (Table 1). Among these 14, a total of 11 were identified retrospectively, including two in 2012, five in 2014, and four in 2015. Three were identified prospectively in 2016 after the MDPH request, the most recent in late July 2016. None of the reports of patients with onset before 2012 met the clinical case definition.

**TABLE 1:** Selected characteristics of 14 patients with sudden-onset amnesia and complete hippocampal ischemia of unclear etiology — Massachusetts, June 2012–July 2016

Characteristic	No. (%)
**Age group (yrs)**	
19–30	6 (43)
31–40	2 (14)
41–52	6 (43)
**Male sex**	**10 (71)**
**Reported history of substance use**	**13 (93)**
Opioids*	12 (86)
Benzodiazepines^†^	6 (43)
Marijuana	6 (43)
Cocaine	5 (36)
Amphetamines (dextroamphetamine/amphetamine)	2 (14)
Lysergic acid diethylamide (LSD)	2 (14)
3,4-methylenedioxymethamphetamine (MDMA)	1 (7)
Mushrooms	1 (7)
Phencyclidine (PCP)	1 (7)
**Toxicology screening (blood and/or urine) done**	**13 (93)**
Any positive results	11 (85)
Opiates	8 (62)
Marijuana/Cannabinoids	4 (31)
Cocaine	2 (15)
Benzodiazepines	2 (15)
Amphetamines	1 (8)
Barbiturates^§^	1 (8)
Salicylates	1 (8)
Multiple substances	5 (38)

* One patient had oxycodone/acetaminophen prescribed.

^†^ Four patients had a benzodiazepine prescribed.

^§^ Patient had butalbital/acetaminophen/caffeine prescribed.

All 14 patients had been evaluated at hospitals in eastern Massachusetts. One was a resident of southeastern New Hampshire and another was visiting from the state of Washington. Patient age ranged from 19 to 52 years (mean and median = 35 years). Nine patients were unconscious at the time they came to medical attention, five of whom required endotracheal intubation. After regaining consciousness, all nine were noted to be amnestic. Among the other five patients, family members, friends, or acquaintances observed the emergence of severe memory loss after limited time apart and brought them to the emergency department for further assessment. In addition to striking anterograde amnesia, deficits of orientation, attention, and executive function were variously noted. These deficits were reported to have improved over time, with resolution of memory loss in one patient at 5 months, but persisting in two patients with follow-up of more than 1 year (Table 2). For 13 patients, MRI of the head was performed within 5 days of initial evaluation, and at 8 days in the 14th patient. In addition to bilateral hippocampal ischemia (Figure), nine patients also exhibited ischemic changes in one or more, often asymmetric extra-hippocampal regions, primarily in the subcortical and posterior areas (Table 2). Follow-up MRI in one patient, at 5 weeks, demonstrated complete resolution of the initial abnormalities; in two other patients, at 13 and 22 months after onset, MRI revealed residual, bilateral hippocampal volume loss.

**TABLE 2:** Selected characteristics of 14 patients with sudden-onset amnesia and complete hippocampal ischemia of unclear etiology, by onset year — Massachusetts, June 2012–July 2016

Onset year	Age (yrs)	Sex	Substance abuse disorder history	Positive toxicology results	Locations of extra-hippocampal signal abnormalities on MRI	Clinical follow-up
2012	27	M	Opioids, marijuana	Opiates	None	Not available
2012	22	M	Opioids, marijuana, LSD, MDMA, cocaine	Opiates	None	At 22 months, residual mildly impaired attention and storage, variable processing speed
2014	49	M	None reported	Opiates, cocaine	Occipital lobe	Not available
2014	21	M	Opioids	Marijuana	Basal ganglia, fornix, midbrain, cerebellum, temporal lobe	Not available
2014	51	F	Opioids, marijuana, cocaine	Opiates,* cannabinoids, salicylates	Cerebellum, occipital lobe	Not available
2014	33	F	Opioids (benzodiazepine prescribed)	Opiates, benzodiazepine	Basal ganglia	At 13 months: moderate short-term memory loss, mild inattention and executive dysfunction (for visuospatial and language tasks)
2014	41	M	Opioids	Not performed	None	At 8 weeks: severe short-term memory loss, mildly diminished working memory; at 9 months: died from cardiac arrest
2015	46	M	Opioids (benzodiazepine prescribed)	Negative	None	Not available
2015	19	M	Marijuana, LSD, mushrooms, amphetamine/ dextroamphetamine	Cannabinoids	Cerebellum	At 5 months: short-term memory loss resolved; persistent seizure disorder
2015	52	F	Opioids, cocaine (benzodiazepine prescribed)	Opiates, barbiturates^†^	Basal ganglia	Not available
2015	36	M	Opioids	Negative	Basal ganglia, corpus callosum, centrum semiovale, occipital lobe, cerebellum	Not available
2016	21	F	Opioids, cocaine, benzodiazepine, marijuana	Opiates	Basal ganglia	Not available
2016	22	M	Opioids, benzodiazepine, marijuana (benzodiazepine prescribed)	Marijuana	None	Not available
2016	50	M	Opioids, benzodiazepine, PCP, cocaine, amphetamine/ dextroamphetamine	Amphetamines, benzodiazepine, cocaine, opiates	Parietal lobe	Not available

**Abbreviations:** F = female; LSD = lysergic acid diethylamide; M = male; MDMA = 3,4-methylenedioxymethamphetamine; MRI = magnetic resonance imaging; PCP = phencyclidine.

* Patient had oxycodone/acetaminophen prescribed.

^†^ Patient had butalbital/acetaminophen/caffeine prescribed.

A history of substance use disorder was documented in 13 of 14 patients; the remaining patient tested positive for opiates and cocaine at the time of initial evaluation. The other patient, who tested positive for cocaine, also tested positive for opiates, amphetamines, and benzodiazepines, none of which were being prescribed at the time. Overall, 12 of 14 patients had a history of opioid use, and eight tested positive for opiates on routine toxicology screening, including one whose medication list included oxycodone-acetaminophen and another who had not reportedly filled a prescription for buprenorphine/naloxone in approximately 2 months. Among the six patients with a history of benzodiazepine use, four had lorazepam or clonazepam on their medication list, and two tested positive for benzodiazepines. Tobacco and alcohol histories were incompletely documented for multiple patients, although no patient tested positive for alcohol on routine screening. One of the two patients with negative toxicology results upon routine testing had reported heroin use in the days preceding the event, and the other had a history of opioid use, but further details were unavailable. Marijuana, lysergic acid diethylamide (LSD), 3,4-methylenedioxymethamphetamine (MDMA, commonly known as Ecstasy), mushrooms, and phencyclidine (PCP) were among other substances reported to have been used (Table 2). Neither of the patients with a history of dextroamphetamine/amphetamine use had amphetamines listed as a prescribed medication. Among four patients with gabapentin on their active medication list, one reportedly had evidence of gabapentin overdose at the time of evaluation. Routine clinical toxicology screening in that patient also revealed the presence of opiates, cannabinoids, and salicylates.

One patient had a history of seizures on two occasions in the past, possibly related to alcohol withdrawal, but no evidence of seizure at the time of assessment. Another patient had witnessed seizure activity during transport to the emergency department, but had no history of seizures. A third patient developed a seizure disorder after evaluation for the amnestic episode. No epileptiform abnormalities were noted on electroencephalography (EEG) at the time of initial evaluation in these three patients or in eight others who underwent EEG.

Six patients had history of at least one vascular disease risk, including hypertension, dyslipidemia, diabetes, and sleep apnea. Echocardiogram performed in six patients, and vessel imaging of the head and neck performed in seven patients, did not reveal a source of thromboembolism. Electrocardiogram revealed a new diagnosis of atrial fibrillation in the two oldest patients (aged 50 and 52 years). One patient aged 36 years demonstrated pulseless electrical activity and respiratory arrest (after a documented brief response to naloxone), with resolution on prehospital resuscitation. Cerebrospinal fluid findings in five patients who underwent lumbar puncture were unremarkable. Carboxyhemoglobin and methemoglobin levels were measured in two patients and were unremarkable. Initial aspartate and alanine aminotransferase were elevated in all 13 patients tested, with both levels in one patient exceeding 500 units/liter (approximately 10 times the upper limit of normal). Otherwise, extensive work-up was unremarkable. Investigation of the 14 cases is ongoing.

## Discussion

The combination of clinical findings described in this report has previously been reported rarely and in isolation, associated with isolated cocaine use, influenza, and carbon monoxide poisoning (*2*–*6*). This cluster of amnestic syndrome associated with bilateral complete hippocampal ischemia is unusual given the absence of a readily identifiable etiology, the temporospatial clustering, relatively young patient age, and extensive substance use among affected persons.

Cardiopulmonary, cerebrovascular, or other mechanisms might serve as plausible explanations underlying certain findings. Hypoxemic injury to the relatively vulnerable hippocampal regions, for example, has been raised as one possibility (*10*). However, further case identification and reporting are needed to determine whether these combined observations represent an emerging syndrome related to substance use or other causes (e.g., a toxic exposure).

The findings in this report are subject to at least three limitations. First, information was obtained from medical records from several different facilities, and differences in documentation and medical assessment across patients limited the consistent characterization of variables. Second, this investigation was intended to establish the existence of the case cluster and generate hypotheses about possible associated exposures. A case-control study could more rigorously test potential associations. Finally, the identification of cases required that MRI of the head had been performed during patient work-up, which might not be consistently performed by medical providers for various reasons.

MRI of the head, toxicology screening, and neurologic consultation should be considered in all adults aged ≥18 years with sudden-onset amnesia, particularly in patients with altered consciousness. Advanced laboratory testing, including testing for synthetic opioids (e.g., fentanyl) and their analogues, as well as extraneous substances not assessed in these reported cases, might further clarify an association with substance use.

**Summary**

**What is already known about this topic?**

Acute, complete, and bilateral ischemia of the hippocampus is a rare cause of memory loss (associated with toxic exposure, among other etiologies) that has been reported rarely and in isolation. A single 2013 case of complete unilateral hippocampal ischemia has been linked to heroin inhalation.

**What is added by this report?**

A unique cluster of 14 cases of sudden onset amnesia with acute, complete, and bilateral ischemia of the hippocampus was identified in Massachusetts during 2012–2016. No clear etiology exists, but at time of initial evaluation, 13 of 14 tested positive for opioids or had opioid use recorded in their medical history.

**What are the implications for public health practice?**

The apparent temporospatial clustering, relatively young age at onset (19–52 years), and extensive substance use associated with this group of patients suggests broader surveillance is needed to determine whether this represents an emerging syndrome related to substance use or other causes, including introduction of a toxic substance.
